# The application of fNIRS-sEMG in the study of muscle-brain coupling

**DOI:** 10.3389/fneur.2026.1879060

**Published:** 2026-06-19

**Authors:** Tinghui Zhang, Ji Zhang, Lin Gao, Yujing Zhang, Wanchao Zhang, Pengbin Zhao, Xiantao Tai

**Affiliations:** The Second Clinical Medical College, Yunnan University of Chinese Medicine, Kunming, China

**Keywords:** cortical muscle coherence, fNIRS, muscle-brain coupling, review, sEMG

## Abstract

With the rapid development of functional near-infrared spectroscopy and surface electromyography, combining the two offers a new way to accurately evaluate muscle-brain coupling, with unique advantages for research on real motor control and neural rehabilitation. This paper analyzes the principle of combined technology, commonly used coupling indicators, and their application in nervous system diseases, motor control in healthy people, and chronic pain diseases. After summarizing, it is found that there is a stable and repeatable functional coupling relationship between specific cortical regions and specific muscle groups. A significant reduction in cortical-muscle coupling accompanied compensatory cortical hyperactivation. The coupling laws of different populations are different. Under the state of exercise in healthy people, the specific functional brain areas, such as the primary motor cortex and primary sensory cortex, can form a stable and repeatable specific functional coupling relationship with the corresponding muscle groups of the body, and the coupling strength is highly matched with movement accuracy, muscle force output, and motor coordination. Patients with nervous system diseases have typical characteristics of abnormal neural remodeling, which are generally manifested as compensatory overactivation of specific cortex and disturbed resource allocation in brain regions, accompanied by multiple pathological features such as significant attenuation of cortical-muscle coupling efficiency, disturbance of coupling rhythm, and decreased synchrony. Patients with chronic pain have abnormal regulation of brain areas and an imbalance of muscle activation. Due to the difference in sampling rate between fNIRS and sEMG, there are some challenges in signal processing, and there is no unified and standardized signal processing and data analysis system, which limits the versatility and standardization of this technique to a certain extent. However, fNIRS-sEMG is still one of the preferred methods to evaluate the muscle-brain coupling at present.

## 1 Introduction

Muscle-brain coupling, also defined as corticomuscular coupling, refers to the functional interaction between the cerebral cortex (especially motor-related regions) and peripheral muscles. It represents the bidirectional interaction and functional integration between the central nervous system and the skeletal muscle system for precise motor control (1). Corticomuscular coherence (CMC) is a classical frequency-domain indicator for evaluating the strength of muscle-brain coupling, which is utilized to quantify the synchronization level between cortical oscillations and muscle activities. In certain research scenarios, it characterizes the concept of muscle-brain coupling and reflects the frequency-domain synchronization of descending motor commands and ascending sensory feedback between the motor cortex and skeletal muscles, with the beta band (13–30 Hz) serving as the core frequency band (2). The quantification of the brain’s motor command output, sensory feedback perception, and bidirectional information flow can be achieved through the calculation of corticomuscular coherence, transfer entropy, Granger causality analysis, and correlation coefficients, thereby reflecting the degree of muscle-brain coupling (3, 4). A high level of muscle-brain coupling is considered a manifestation of refined and efficient neural drive and sensory feedback integration between the motor cortex and target muscles, which indicates favorable motor control performance. Stable and repeatable functional coupling exists between specific cortical regions and muscle groups, and consistent decoupling patterns can be observed under pathological conditions. The decoupling characteristics combining compensatory overactivation of the cortex and significantly reduced corticomuscular coupling are commonly identified in the shared pathological phenotypes of neuromuscular dysfunction. Additionally, a time delay in muscle-brain coupling is detected under pathological conditions, suggesting universal impairment of neural conduction efficiency (5).

Functional near-infrared spectroscopy (fNIRS) is a non-invasive optical imaging technique based on the principle of neurovascular coupling (6, 7). The device is lightweight and has strong anti-interference ability to head motion artifacts. The ability to maintain stable signals during natural dynamic tasks fits the research needs of real motor control and neural rehabilitation (8, 9). Surface Electromyography (sEMG) is widely used in sports biomechanics, rehabilitation medicine, neuroscience, and human-computer interaction interfaces due to its advantages of non-invasive, easy operation, and dynamic recording of multi-channel muscle activity (10). The combination of fNIRS and sEMG can simultaneously obtain the hemodynamic response of cerebral cortex and the electrical activity characteristics of muscle, to evaluate the spatiotemporal dynamic changes of neurovascular coupling and neuromuscular coupling in real time in the natural movement environment (11), providing a real-time evaluation method for the study of neuromuscular coupling.

**Figure 1:**
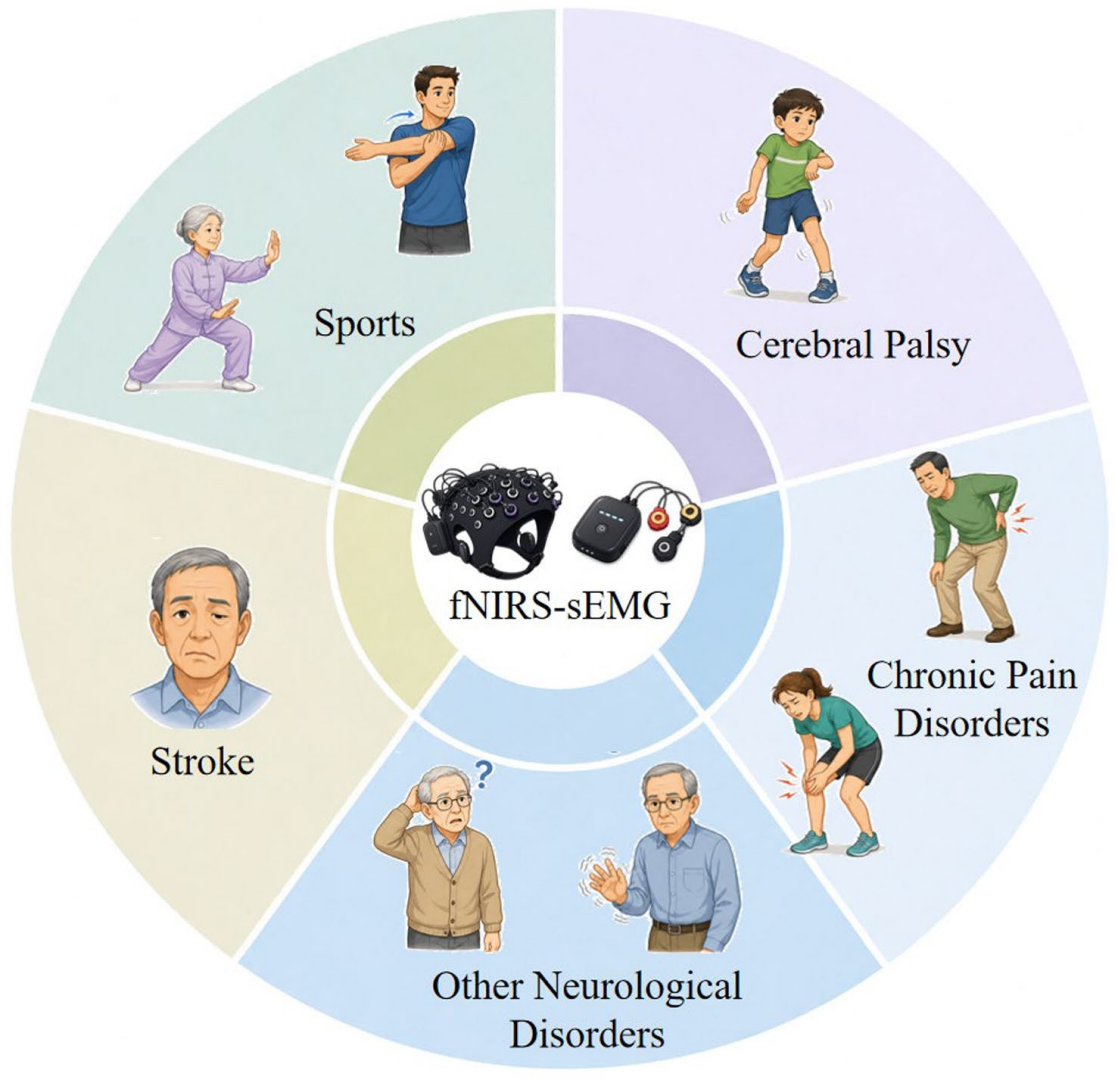
Graphical abstract.

Existing reviews on the combined use of fNIRS and sEMG techniques have primarily focused on single disease entities or methodological aspects, lacking a systematic integration across diverse populations and multiple clinical/functional scenarios. Previous investigations of corticomuscular coupling have predominantly employed EEG-sEMG paradigms. The systematic review by Seynaeve et al. (12) comprehensively synthesized evidence from studies using simultaneous EEG-sEMG recordings during gait, confirming the presence of robust corticomuscular coupling during walking in healthy individuals. This coupling strength is modulated by walking speed, task complexity, and environmental conditions, with coupling analyses primarily relying on coherence measures in the beta and gamma frequency bands. With advances in signal processing techniques, challenges in sampling synchronization between fNIRS and sEMG have been progressively addressed, allowing coherence metrics originally established for EEG-sEMG paradigms to be extended to fNIRS-sEMG combined systems. Compared with prior work, the present review provides a comprehensive synthesis of the applications of fNIRS-sEMG integration across multiple scenarios, including neurological disorders, chronic pain conditions, and motor control in healthy populations. It further systematically summarizes the characteristics and patterns of muscle-brain coupling across different populations (Figure 1).

## 2 Principle of fNIRS-sEMG combination technology

### 2.1 fNIRS technique

Functional near-infrared spectroscopy (fNIRS) is an optical neuroimaging technique based on the neurovascular coupling principle. It emits near-infrared light with specific wavelengths ranging from 700 to 900 nm and calculates real-time changes in regional cerebral oxygen saturation according to the differences in light absorption coefficients between oxygenated hemoglobin (HbO) and deoxygenated hemoglobin (HbR). Its core mechanism relies on regional cerebral hemodynamic responses induced by neural activation. Specifically, the initial neural metabolic demands triggered by the activation of specific brain regions lead to a transient reduction in HbR concentration, followed by a pronounced increase in HbO concentration due to vasodilation and the influx of fresh blood (13). By capturing such hemodynamic responses with a time lag of approximately 1–2 s, fNIRS enables the characterization of activation patterns and network connectivity features of specific brain regions under various task conditions.

Benefiting from its non-invasiveness and strong resistance to motion artifacts, fNIRS exhibits unique advantages in dynamic scenarios, including force maintenance, slow fine motor movement, and rehabilitation training (6). Unlike electroencephalography (EEG), which is highly sensitive to variations in scalp electrical resistance, fNIRS is minimally affected by scalp motion interference. Additionally, its portable and lightweight equipment allows fNIRS to be applied in real-life scenarios such as walking and running (8).

### 2.2 sEMG technique

Surface electromyography (sEMG) is a non-invasive detection technique that records bioelectrical signals generated during muscle contraction via surface electrodes attached to the skin (14). Its fundamental principle originates from the electrical activity of motor units. When motor neurons discharge, the innervated muscle fibers contract synchronously and produce subtle electrical potentials, which propagate through body fluids and cutaneous tissues to the skin surface electrodes (10). The amplitude of sEMG signals is positively correlated with muscle contraction intensity, while frequency-domain features such as the median frequency (MDF) can reflect the degree of muscle fatigue and the recruitment patterns of motor units (15). Unlike intramuscular electromyography, sEMG exhibits prominent advantages, including non-invasiveness, convenient operation, and the capability of dynamically recording multi-channel muscular activities. Accordingly, it has been widely applied in multiple research fields, including sports biomechanics, rehabilitation medicine, neuroscience, and human–computer interaction (16). Through time- and frequency-domain analyses, researchers can objectively quantify muscle activation patterns, evaluate neuromuscular disorders, and monitor dynamic alterations in muscle function during training and therapeutic interventions.

Time-domain analysis primarily focuses on the temporal waveform variations of sEMG signals and characterizes muscular electrical activity by calculating signal statistical features. Root mean square (RMS) represents the most widely adopted time-domain parameter. It reflects the root square of signal energy, can directly quantify the intensity of muscle contraction, and exhibits a strong linear correlation with muscle-generated torque. Additionally, integrated EMG and mean absolute value (MAV) serve as classic time-domain indicators (17). These parameters are capable of evaluating the overall magnitude of muscular activity and are commonly utilized to assess training intensity in athletes and monitor rehabilitation progress in patients with motor dysfunction.

In contrast, frequency-domain analysis concentrates on the energy distribution of sEMG signals across different frequency components and is predominantly applied to evaluate muscle fatigue and motor unit recruitment patterns. During sustained muscle contraction, the electrical conduction velocity of muscle fibers decreases, resulting in the attenuation of high-frequency components and the enhancement of low-frequency components. Accordingly, median frequency (MDF) and mean frequency (MNF) are recognized as core parameters for assessing muscular fatigue. Such alterations in frequency-domain characteristics reflect increases in the membrane potential threshold of motor units and adaptive modifications in motor unit firing patterns (18).

### 2.3 Advantages of fNIRS-sEMG combination

The integrated application of fNIRS and sEMG enables synchronous observation of central and peripheral neural activities, namely the multi-scale synchronous recording of cerebral hemodynamic responses and neuromuscular electrical activities. fNIRS captures cortical hemodynamic responses to reflect neuronal metabolic demands (19), while sEMG records peripheral electrical activities of muscle fibers to reveal motor unit recruitment patterns and the intensity of muscle activation. Such full-chain monitoring capability of muscle-brain interactions cannot be achieved by single-modality techniques.

Furthermore, the objectivity and sensitivity of the fNIRS-sEMG combination have been validated in clinical rehabilitation assessments. Existing studies have demonstrated that multimodal fNIRS-sEMG recordings can sensitively detect impairments in muscle-brain coupling. Multiple quantitative indicators, including correlation coefficients, corticomuscular coherence, and transfer entropy (1, 20), allow for precise quantification of corticomuscular coupling characteristics before and after therapeutic interventions, thereby supporting the evaluation of rehabilitation outcomes. This multimodal approach enables clinicians to accurately assess the progression of neural plasticity and formulate individualized and targeted rehabilitation strategies.

## 3 Common coupling index

In muscle-brain coupling research, fNIRS indirectly reflects cortical neural activation by detecting dynamic changes in cerebral oxygenation, while sEMG directly characterizes peripheral motor output by recording muscular electrical activity. A high coupling intensity indicates effective cortical modulation of muscular activation and adaptive regulation of cortical oscillations by sensory afferent inputs. Such quantitative features provide objective evidence for elucidating the mechanisms of sensorimotor integration, as well as plastic alterations during motor learning and rehabilitation.

**Figure 2:**
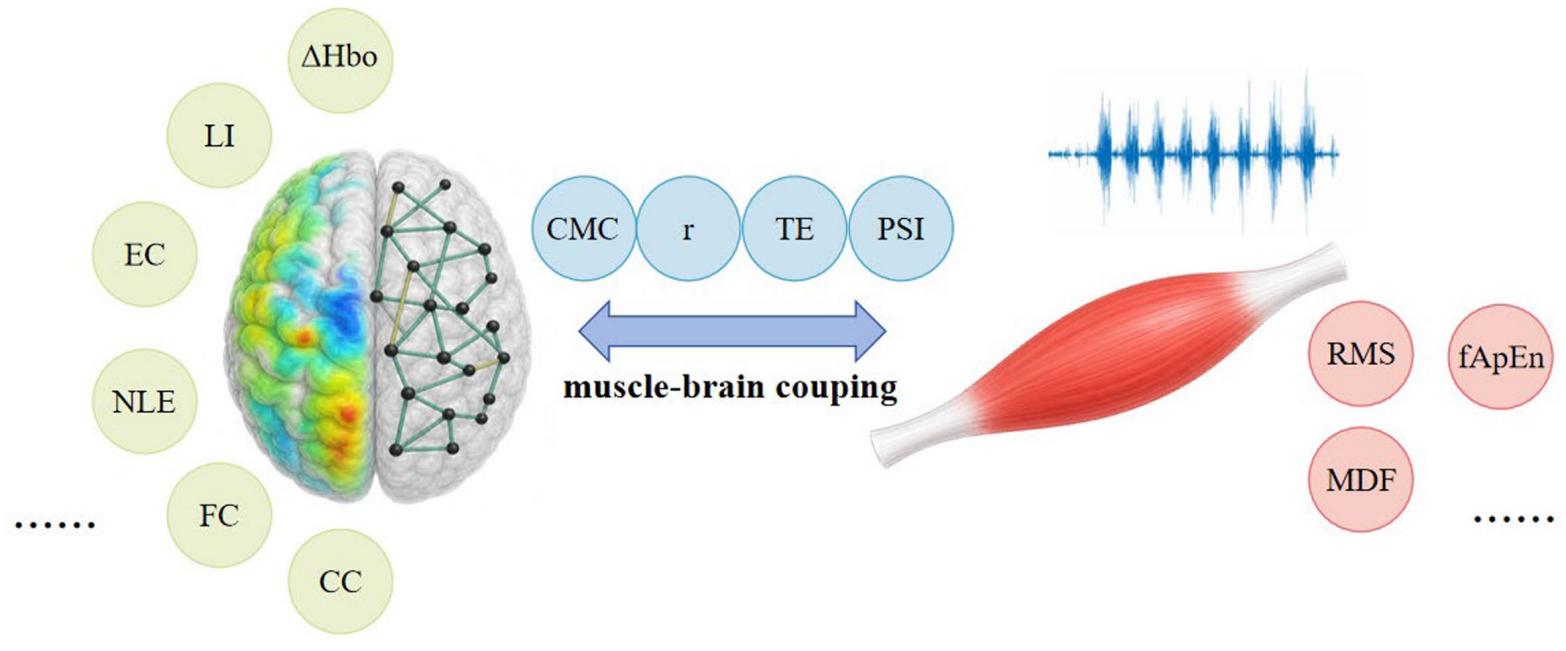
Schematic of muscle-brain coupling metrics based on fNIRS-sEMG.

Core indicators for evaluating corticomuscular coupling can be categorized into three classes: cortical activation indicators, muscular activity indicators, and cross-modal coupling indicators. Cortical activation indicators primarily include ΔHbO concentration in multiple brain regions (e.g., primary motor cortex, primary somatosensory cortex, supplementary motor area, premotor cortex, prefrontal cortex, dorsolateral prefrontal cortex, and occipital lobe), laterality index, functional connectivity strength, effective connectivity strength, as well as topological properties of brain networks such as small-world attributes, nodal local efficiency, and clustering coefficient (19). Muscular activity indicators mainly encompass RMS, MDF, fuzzy approximate entropy (fApEn), co-contraction index (CCI), coefficient of variation (Cv), and synergy stability index (SSI) (21). Cross-modal coupling indicators consist of transfer entropy (TE), Pearson correlation coefficient (r), corticomuscular coherence (CMC), and phase synchronization index between cortical hemodynamic signals and peripheral sEMG signals (22) (see Table 1 and Figure 2 for details).

**Table 1:** Summary of relevant literature.

References	Participants	Task	fNIRS target brain regions	sEMG target muscles	Indicators
Hervey et al. (27)	Cerebral palsy	Unimanual finger tapping	PMC, SMA, M1, S1	FDS, EDC, BB, TB	r, AT
Cheng et al. (23)	Stroke	Robotic training	PFC, SFC, SMA, PMC	FCR, FDS, FCU, APL	RMS, ΔHbO, r
Wang et al. (11)	Stroke	Elbow flexion and extension	PFC, MC, OL	BB, TB	ΔHbO, r, fApEn, TE
Wei et al. (24)	Stroke	Isometric wrist extension	PFC, SMA, M1	ECU, FCR	ΔHbO, RMS, fApEn, FC, MF, PSI, CMC
Yang et al. (25)	Post-stroke fallers	Simple/difficult sit-to-stand	M1, SMA, DLPFC	RF, BF	ΔHbO, FC, MA
Ye et al. (26)	Stroke	Bilateral elbow flexion	PMC, M1, S1	DA, DP, BI, TI, BIO	ΔHbO, LI, SSI, Cv, CT
Fu et al. (30)	mild cognitive impairment	Posture-cognition dual tasks	DLPFC, SMA, M1	BTAM	ΔHbO, RMS, CCI, r
Yang et al. (41)	Chronic low back pain patients	Single-leg standing	DLPFC, M1, S1, SMA	TrA, SLM, TA, GM	ΔHbO, RMS, FC, CCI, r
Deng et al. (34)	Knee osteoarthritis	Level walking/stair ascent and descent	M1, S1, SAC	RF, VM, VL, BF, TA, GM, GL	ΔHbO, RMS, MDF
Li et al. (32)	Older adults	Musical Tai Chi	DLPFC, FPC	GM, VL, AO, ES, MD, BB	ΔHbO, RMS, FC, CMC, MDF
Wang et al. (31)	Young adults	Isometric elbow contraction	PFC, MC, OL	BIC, TRI	ΔHbO, FCM, fApEn, CCI, EC, CC, NLE, r

Comparison of terms: PMC, Premotor Cortex; SMA, Supplementary Motor Area; M1, Primary Motor Cortex; S1, Primary Somatosensory Cortex; PFC, Prefrontal Cortex; SFC, Superior Frontal Cortex; MC, Motor Cortex; OL, Occipital Lobe; DLPFC, Dorsolateral Prefrontal Cortex; SAC,association cortex; FCR, flexor carpi radialis; FDS, flexor digitorum superficialis; EDC, extensor digitorum communis; BB, biceps brachii; TB, triceps brachii; FCU, flexor carpi ulnaris; APL, abductor pollicis longus; ECU, extensor carpi ulnaris; RF, rectus femoris; BF, biceps femoris; DA, deltoid anterior; DP, deltoid posterior; BI, brachialis; TI, tibialis posterior; BIO, biceps brachii; TrA, transversus abdominis; SLM, semimembranosus; TA, tibialis anterior; GM, gluteus maximus; VL, vastus lateralis; AO, abdominal oblique; ES, erector spinae; MD, medial deltoid; BIC, biceps brachii; TRI, triceps brachii; BTAM, bilateral trunk and ankle muscles; VM, vastus medialis; GL, lateral head of gastrocnemius; RMS, Root Mean Square; ΔHbO, Change in Oxygenated Hemoglobin; fApEn, Fuzzy Approximate Entropy; FC, Functional Connectivity; MF, Median Frequency; PSI, Phase Synchronization Index; CMC, Cortico-Muscular Coherence; LI, Laterality Index; SSI, Synchronization Strength Index; Cv, Coefficient of Variation; CT, Contour Temporal Similarity; CCI, Co-Contraction Index; EC, Effective Connectivity; CC, Clustering Coefficient; NLE, Nodal Local Efficiency; MA, Mean Amplitude; AT, Activation Timing.

## 4 Application

### 4.1 Stroke

Cheng et al. (23) combined fNIRS and sEMG to systematically explore the specific correlations between forearm muscle activation and cortical activation during robot-assisted training in 10 patients with upper limb dysfunction following subacute stroke. The results revealed distinct muscle-brain coupling patterns across the three training protocols. Passive training predominantly activated the premotor cortex (PMC), with a moderate positive correlation between the RMS of forearm muscles and ΔHbO (*r* = 0.4659). This pattern was characterized by synergistic activation of flexor muscles without evident task specificity. Mirror training induced pronounced activation of the prefrontal cortex (PFC) and superior frontal cortex (SFC). Significant positive correlations were observed between the RMS of grip-related muscles, including the flexor carpi radialis (FCR) and flexor digitorum superficialis (FDS), and ΔHbO (*r* = 0.6387), suggesting improved cognitive-motor integration. Resistance training yielded the strongest activation in the supplementary motor area (SMA) and PMC. A strong positive correlation was found between the RMS of resistance muscles [flexor carpi ulnaris (FCU) and adductor pollicis (AP)] and ΔHbO (*r* = 0.6982). Inter-group comparison further indicated that the RMS of target muscles during resistance training was significantly higher than that during mirror training. After three training sessions, patients’ Motor Status Scale scores increased significantly from 41.8 ± 23.4 to 46.7 ± 22.4 (*p* = 0.001). This study demonstrates that different robot-assisted training paradigms modulate neural plasticity via differentiated coupling between specific muscles and corresponding cortical regions. Specifically, resistance training facilitates strong coupling between the SMA–PMC network and wrist flexors as well as thumb abductors, while mirror training strengthens the coordination between the PFC–SFC network and grip muscles.

Wei et al. (24) utilized fNIRS-sEMG integration to investigate corticomuscular coupling characteristics during 50% MVC isometric wrist extension in patients with subacute stroke. The results demonstrated significantly decreased coherence and phase synchronization between the left primary motor cortex (M1)/extensor carpi ulnaris (ECU)-flexor carpi radialis (FCR), left SMA/ECU-FCR, and left PFC/FCR in patients. fNIRS measurements revealed elevated ΔHbO concentrations in the bilateral SMA, M1, and right PFC, accompanied by enhanced functional connectivity of RSMA–RPFC, RSMA–LPFC, and LM1–RPFC. Meanwhile, patients exhibited lower ECU RMS, as well as reduced MDF and fApEn of the FCR and ECU compared with healthy controls. This study identified a dissociative neuro-muscular pattern in subacute stroke patients, characterized by excessive cortical activation and strengthened intracortical connectivity, alongside attenuated corticomuscular coupling and decreased muscular activity complexity.

Wang et al. (11) adopted fNIRS-sEMG integration to characterize neuromuscular coupling in stroke patients during elbow movement at 30 and 70% maximum voluntary contraction (MVC). sEMG signals from the biceps brachii (BB) and triceps brachii (TB) and HbO₂ signals from the bilateral prefrontal cortex (PFC), motor cortex (MC), and occipital lobe (OL) were synchronously acquired. At identical contraction intensities, stroke patients exhibited significantly lower fuzzy approximate entropy (fApEn) of the affected agonists and antagonists than healthy controls, and the fApEn of affected agonists was positively correlated with Fugl-Meyer Assessment (FMA) scores (BB during flexion: *r* = 0.6593, *p* = 0.0197; TB during extension: *r* = 0.6475, *p* = 0.0228). fNIRS results indicated reduced activation in the contralesional motor cortex (CL MC) and compensatory ipsilesional motor cortex (IMC) activation in patients. The ipsilesional effective connectivity toward the CL MC was positively correlated with FMA scores during both flexion (*r* = 0.5935, *p* = 0.0419) and extension (*r* = 0.6542, *p* = 0.0210). Corticomuscular coupling analysis revealed decreased transfer entropy (TE) from the CL MC to affected-side agonists, and CL MC-BB TE at 70% MVC flexion was positively associated with FMA scores. Moreover, patients showed significantly lower brain network small-worldness and CL MC nodal local efficiency than healthy individuals. This study demonstrates that stroke induces reduced muscular electrical complexity of the BB and TB, as well as impaired bilateral motor network efficiency and corticomuscular information transmission.

Yang et al. (25) adopted fNIRS-sEMG multimodal recording to explore the associations between cortical-muscular activation patterns and fall risk during sit-to-stand tasks in stroke patients. With increasing task difficulty, stroke patients exhibited progressively increased muscular activation on the affected side, with overall higher activation amplitudes than healthy controls, whereas no significant differences in muscle activation were found between high- and low-fall-risk subgroups. During task escalation, patients showed further elevated ΔHbO in the unaffected SMA and dorsolateral prefrontal cortex (DLPFC) but failed to enhance interregional functional connectivity, which was significantly improved in healthy individuals. Intergroup comparisons revealed that patients exhibited stronger activation in the unaffected SMA and DLPFC than healthy controls under both simple and difficult tasks (simple task: SMA, *p* = 0.016; DLPFC, *p* = 0.047; difficult task: SMA, *p* = 0.001; DLPFC, *p* < 0.001). Additionally, patients with high fall risk presented higher unaffected SMA activation during simple sit-to-stand tasks than low-risk patients. This study demonstrates that stroke patients manifest an inefficient cortical resource utilization pattern characterized by excessive cortical activation with insufficient functional connectivity.

Ye et al. (26) applied fNIRS-sEMG integration to investigate muscle synergy patterns and interhemispheric activation balance during bilateral elbow flexion in stroke patients, and further established a quantitative evaluation model for upper limb motor function. Compared with healthy controls, stroke patients exhibited significantly lower vector cosine (Cv) and synergy stability index (SSI) of the affected limb muscles, as well as a reduced laterality index (LI) of the PMC and M1 during affected limb movement, indicating abnormal muscle synergy organization and disrupted interhemispheric activation balance. Further regression analysis demonstrated that a linear model integrating four indicators (affected-side Cv, ipsilateral/contralateral Cv ratio, unaffected-side PMC LI, and affected/unaffected maximum voluntary contraction ratio) effectively predicted upper-limb Fugl-Meyer Assessment scores (*R*^2^ = 0.860, *p* < 0.001). In contrast, single indicators showed limited predictive performance.

### 4.2 Cerebral palsy

Hervey et al. (27) adopted fNIRS-sEMG to develop GLM/ICA approaches for finger-tapping tasks in children with hemiplegic cerebral palsy (CP). Subject-specific regressors were constructed based on linear envelopes of sEMG signals from finger extensors and flexors. Combined with independent component analysis of ΔHbO signals, this method distinguished cortical activation associated with voluntary versus mirror movements. The results revealed substantial contamination of fNIRS signals during mirror movements, with correlation coefficients of 0.59–0.78 between regressors of the two movement types; accordingly, 25–50% of independent components were excluded due to crosstalk. The sEMG regressors yielded correlations of 0.72–0.96 and enabled the detection of covert muscle activation invisible to visual inspection. After correction, spurious activation in the ipsilateral M1/S1 overestimated by the conventional Boxcar-GLM was eliminated, yielding physiologically reasonable activation patterns.

Zhou et al. (28) combined fNIRS with Vicon three-dimensional motion capture to explore the relationship between lower limb joint coordination and cortical activation during walking in adolescents with spastic CP. The CP group exhibited higher joint coordination variability (DP) of hip-knee and knee-ankle joints than healthy controls, while no intergroup difference was found in the mutual angular correlation relative phase (MACRP). Meanwhile, ΔHbO levels in the bilateral SMA, precentral gyrus (PRG), postcentral gyrus (POG), and superior parietal lobule (SPL) were markedly elevated in patients. Correlation analyses showed that hip-knee DP was strongly positively correlated with bilateral SPL (Ch9, 18) and right POG (Ch17), and knee-ankle DP was positively associated with right POG (Ch17) in the patient group. In healthy subjects, knee-ankle MACRP correlated positively with right PRG/POG (Ch15), hip-knee DP correlated positively with right SMA (Ch12), and negatively with left SPL (Ch9). This study indicates that reduced intersegmental coordination stability of lower limbs during walking in patients with cerebral palsy is accompanied by widespread overactivation of the sensorimotor cortex.

Excessive activation of the SPL and primary somatosensory cortex (S1) is closely linked to increased gait variability. Jochumsen et al. (29) used combined EEG-sEMG recordings to investigate motor intention detection in eight adolescents with CP during ankle dorsiflexion. They acquired 9-channel EEG and tibialis anterior sEMG signals and established a random forest classification model to identify optimal electrode layouts and feature types. sEMG signals were used to label movement onset and distinguish motor intention from resting states, with time-domain, frequency-domain, and template-matching features extracted for classification. The results showed classification accuracies of approximately 70% for single-channel recordings, 80% for channels with large Laplacian filtering, and 75% for the full 9-channel set. Time-domain features and multi-feature fusion achieved the best performance. This study verifies the presence of movement-related cortical potentials in these patients and the feasibility of EEG-based motor intention recognition, providing experimental support for the development of brain-computer interface rehabilitation systems for CP.

Literature review reveals that studies applying combined fNIRS-sEMG to motor function assessment in CP remain limited, which may be attributed to the following factors. Technically, involuntary spasticity and abnormal postures prevalent in CP patients induce subtle head movements during tasks, severely degrading fNIRS signal quality. Meanwhile, altered muscle synergy, spastic co-activation, and dystonia render CP-related sEMG signals nonlinear and non-stationary, posing great challenges to time-frequency synchronization of the two modalities. In terms of experimental design, substantial interindividual heterogeneity across CP subtypes and gross motor function levels hinders the establishment of unified acquisition protocols. Such heterogeneity fails to accommodate variations in lesion type, severity, and task load, thereby compromising the validity of group comparisons.

### 4.3 Other neurological disorders

Fu et al. (30) integrated fNIRS, sEMG, and center of pressure (COP) to explore neuromuscular regulation mechanisms during posture-cognition dual tasks in individuals with mild cognitive impairment (MCI). A graded N-back working memory paradigm was adopted for controlled trials. MCI patients exhibited lower cognitive accuracy only under the high-load 2-back task. Under elevated working memory demand, both ankle muscle RMS and body sway magnitude increased, alongside a higher co-contraction index (CCI) throughout the task. fNIRS results revealed excessive activation of bilateral DLPFC, SMA, and M1, as well as reduced DLPFC laterality in MCI patients. Cortical activation was positively correlated with ankle muscle RMS. This study suggests that MCI patients develop cortical overactivation and altered peripheral neuromuscular regulation, which serve as compensatory strategies to maintain postural balance under high cognitive load.

Although single-modal fNIRS or sEMG has been widely applied in neuroscience, few studies have combined the two techniques to investigate muscle-brain coupling in Parkinson’s disease, multiple sclerosis, spinal cord injury, and other neurological disorders. This represents a promising direction for future research.

### 4.4 Motor control in healthy populations

Wang et al. (31) combined fNIRS and sEMG to investigate interactions between muscle activation and cortical network dynamics during isometric elbow flexion at 20 and 80% MVC in healthy adults. Increased contraction strength led to enhanced interhemispheric functional connectivity, particularly between the contralateral PFC, MC, and ipsilateral PFC. Effective connectivity was stronger during movements of the dominant limb relative to the non-dominant limb. Graph theory analyses demonstrated a higher clustering coefficient and nodal local efficiency in the contralateral MC for the dominant limb at 80% MVC compared with 20% MVC. sEMG results showed elevated fApEn and co-contraction index (CCI) of the biceps brachii (BB) and triceps brachii (TB) under high contraction intensity, indicating increased motor unit recruitment and adjusted muscle control strategies. Significant positive correlations were observed between sEMG,fApEn and ΔHbO in the contralateral MC, PFC, and OL. Nodal local efficiency of the contralateral MC for the dominant limb also positively correlated with fApEn. These findings indicate that contraction intensity bidirectionally modulates peripheral muscle activity and central cortical network efficiency, and more efficient corticomuscular information transmission exists for the dominant limb.

Li et al. (32) conducted a 12-week randomized controlled trial to explore the effects of Groovy Oriental Tai Chi (GOTC) on cognitive-emotional integration in older adults. Post-intervention assessments revealed increased RMS and mean power frequency (MPF) of the gluteus medius and vastus lateralis, as well as reduced CCI of the transversus abdominis and multifidus. Meanwhile, ΔHbO in the DLPFC and M1 increased, along with strengthened functional connectivity between these two regions. Correlation analyses showed that changes in M1 ΔHbO were positively correlated with RMS of lower limb muscles (*r* = 0.624, *p* = 0.02) and negatively correlated with trunk muscle CCI (*r* = −0.511, *p* = 0.04), with no obvious lateralization in bilateral lower limbs. This study suggests that GOTC optimizes trunk-limb muscle synergy during postural control in older adults by elevating activation of the DLPFC and M1 and reinforcing their functional connectivity.

### 4.5 Chronic pain disorders

Jiajia et al. (33) adopted combined fNIRS-sEMG to examine the immediate effects of intermittent theta burst stimulation (iTBS) over the left DLPFC on single-leg stance control in patients with chronic low back pain (CLBP). Behavioral assessments were performed before and after iTBS intervention. During right-leg stance, the co-contraction index (CCI) of the right transversus abdominis (TrA) and sacrolumbalis muscle (SLM) decreased significantly, while the RMS of the right gluteus maximus (GM) increased. Meanwhile, activation of the left DLPFC and left M1 was markedly reduced, alongside enhanced functional connectivity between these two regions. Correlation analyses revealed that post-intervention ΔHbO in M1 was negatively correlated with GM RMS (*r* = −0.659, *p* = 0.03) and positively correlated with TrA/SLM CCI (*r* = 0.503, *p* = 0.047). No significant changes were observed during left-leg stance. This study demonstrates that iTBS targeting the left DLPFC optimizes trunk muscle co-contraction and lower limb muscle activation during challenging postural tasks in CLBP patients by attenuating DLPFC and M1 activation and strengthening their cortical connectivity.

Deng et al. (34) designed a cross-sectional study using fNIRS-sEMG to compare sensorimotor cortical activation patterns and central neural regulation between patients with knee osteoarthritis (KOA) and healthy controls during level walking, stair ascent, and descent. A total of 20 KOA patients and 20 healthy participants were recruited. ΔHbO signals from the S1, M1, and sensory association cortex (SAC), as well as sEMG signals from major periarticular knee muscles, were recorded synchronously across the three locomotor tasks. The Visual Analogue Scale (VAS) and Western Ontario and McMaster Universities Osteoarthritis Index (WOMAC) were used to assess pain severity and functional limitation. The research protocol hypothesized aberrant sensorimotor cortical activation in KOA patients during dynamic walking, which may be associated with pain and functional deficits. Previous work has demonstrated hypoactivation of the SAC during isokinetic knee movement in KOA, whereas cortical responses during dynamic walking remain poorly understood. This study aims to clarify corticomuscular coordination across various walking conditions, uncover the underlying central regulatory mechanisms of KOA, and provide theoretical evidence and potential cortical targets for pain management and motor rehabilitation.

## 5 Discussion

A synthesis of the reviewed literature reveals that stable and reproducible functional coupling exists between specific cortical regions and corresponding muscle groups, while patients with neurological disorders commonly exhibit compensatory cortical activation, a dissociative pattern of “high activation with low coupling efficiency”, and delayed activation timing. Notable differences in these coupling characteristics are observed across populations. In healthy individuals, movement tasks elicit stable, reproducible, and functionally specific coupling between key sensorimotor regions (e.g., the primary motor cortex and primary somatosensory cortex) and corresponding body muscles, with coupling strength closely matching motor precision, force output, and movement coordination. In contrast, patients with neurological diseases present typical features of aberrant neural remodeling, generally characterized by compensatory overactivation of specific cortical areas, dysregulated resource allocation, and multiple pathological changes in corticomuscular coupling—including significantly reduced coupling efficiency, disrupted coupling rhythms, and decreased synchronization. Individuals with chronic pain, meanwhile, demonstrate altered cortical regulation and imbalanced muscle activation patterns.

### 5.1 Correspondence of specific cortical regions to specific muscle groups

Based on the anatomical framework of Brodmann Areas (BA), fNIRS enables precise localization and functional differentiation of cortical regions, including the PFC, M1, S1, and PMC (35). For instance, BA4 corresponds to M1, which governs the initiation and regulation of voluntary movement (36). Collectively, the included studies demonstrate stable and reproducible functional coupling between distinct cortical regions and muscle groups, as well as consistent decoupling patterns under pathological conditions.

M1 directly initiates and regulates voluntary movement and controls fine motor activities of the contralateral body (37, 38). Existing evidence indicates that ΔHbO and the effective connectivity strength of M1 are positively and stably coupled with contraction intensity, signal complexity, and motor unit recruitment of upper limb muscles responsible for voluntary control, such as the biceps brachii, triceps brachii, wrist flexors, wrist extensors, and digital flexors. Under pathological conditions, including stroke and chronic pain, the coupling between M1 and voluntary muscles is markedly weakened, manifested as aberrantly elevated cortical activation accompanied by deteriorated muscular regulation efficiency.

S1 receives and processes somatic sensations, including touch, pain, temperature, proprioception, and pressure. It performs preliminary decoding and spatial localization of peripheral sensory signals to provide sensory feedback for motor adjustment (39). Its ΔHbO is positively correlated with proprioceptive input and force stability of muscles for resistance loading and tonic maintenance, such as the biceps brachii, deltoid, gastrocnemius, and soleus. These findings suggest that S1 modulates motor accuracy via sensory feedback, and the sensorimotor loop coupling between S1 and peripheral muscles is substantially impaired in pathological states.

The PMC undertakes motor planning and action selection, integrates multimodal visual and somatosensory information, and facilitates limb coordination and motor learning (40). Its ΔHbO presents stable positive coupling with co-contraction and postural maintenance capacity of proximal stabilizer muscles covering the trunk, pelvis, and lower extremities, including the erector spinae, transversus abdominis, gluteus medius, and quadriceps femoris. The PMC is primarily involved in postural prediction, motor preparation, and multi-muscle coordination, and its coupling with postural stabilizers intensifies with increasing task complexity.

As a core region for higher executive functions, the DLPFC mediates attention modulation, working memory, cognitive control, emotion regulation, motor decision-making, and inter-regional neural integration (41). Its ΔHbO is positively coupled with fine motor control, temporal coordination, and signal complexity of distal hand muscles, such as lumbricals, interossei, flexor digitorum superficialis, and flexor digitorum profundus. The DLPFC participates in motor planning, cognitive engagement, and temporal motor regulation, and its coupling with hand fine motor muscles is significantly enhanced under cognitive-motor dual-task conditions.

The SMA regulates endogenous voluntary movement, sequential action, bimanual coordination, motor inhibition, and rhythmic maintenance, and is also implicated in motor memory and automatic movement control (42). Its ΔHbO is stably positively correlated with synchrony, coordination, and symmetric control of bilateral muscles, including bilateral wrist flexors, quadriceps femoris, and gluteal muscles. The SMA mainly orchestrates bimanual movement sequencing and bilateral muscle synergy, and exhibits stronger coupling with bilateral muscles during symmetric bimanual tasks than during unilateral tasks.

### 5.2 Dissociated pattern of compensatory cortical activation and the “high activation, low coupling” phenomenon

Existing evidence from fNRS-sEMG synchronization studies shows that conditions such as stroke, cerebral palsy, and chronic pain share a common neuropathological phenotype: compensatory cortical overactivation combined with significantly reduced cortico-muscular coupling.

In stroke patients, motor tasks performed with the affected side lead to extensive and excessive activation of bilateral M1, SMA, DLPFC, and PMC. With increasing task difficulty, SMA and DLPFC activity on the healthy side increases further, surpassing levels seen in healthy controls. Despite this high cortical activation, cortex-muscle coherence, phase synchronization, and information transmission efficiency between M1 and target muscles on the affected side are significantly reduced. Consequently, information output from M1 to the muscles is impaired, resulting in inefficient muscle control. Furthermore, while muscle activation amplitude on the affected side may increase compensatorily, parameters such as SSI, Cv, and fApEn decrease, and functional connectivity among brain regions fails to rise with task demands. Thus, an inefficient pattern of ‘high activation, low connectivity, and low coupling’ emerges.

In patients with spastic cerebral palsy, extensive over-activation of bilateral SMA, M1, and PMC during walking is observed. The intensity of this activation is positively correlated with variation in lower limb joint coordination. Despite this increased activity, motor control performance does not improve. Instead, it is associated with abnormal muscle synergy, decreased joint coupling stability, and disordered cortex-muscle closed-loop regulation. These findings suggest that cortical overrecruitment represents a compensatory response rather than adaptive mobilization of neural resources.

In patients with chronic pain, the compensatory activation of DLPFC and M1 is increased during postural control tasks, but the cortex-muscle correlation and regulation efficiency are decreased. Only after neuromodulation, cortical activation decreases, functional connectivity increases, and the muscle synergy pattern is optimized.

In summary, compensatory cortical hyperactivation is not a sign of preserved neural function, but a compensatory response to impaired cortic-muscle coupling and failure of central regulation (43). The dissociation pattern of “high cortical activation, low electromyographic complexity, low cortical-muscle coupling, and low brain network efficiency” can be used as a common neurophysiological feature of neuromuscular disorders such as stroke, cerebral palsy, and chronic pain.

### 5.3 Activation time delay

Time delay is one of the indicators that reflect the central command and peripheral response. In movement disorders caused by brain injury, delays in central-to-peripheral command can result from damage to nerve conduction pathways and decreased synaptic transmission efficiency. Damage to the corticospinal tract directly affects the speed and efficiency with which motor commands are transmitted from the cerebral cortex to spinal motor neurons (44). The neural circuit between the motor cortex, basal ganglia and cerebellum is damaged, affecting the synchronization and accuracy of nerve impulses.

In the above studies, although no literature directly reported time delay, some results could indirectly reflect decreased conduction efficiency. The study by Wei et al. (24) did not directly report the delay value, but the reduction in the phase synchronization index and coherence indirectly reflected the time mismatch in information transmission. Wang et al. (11) found that the TE of the CMC on the affected side of the active muscle was significantly reduced in stroke patients, which also contained delay and directional information.

It should be noted that the low temporal resolution of fNIRS (usually <20 Hz) limits the precise estimation of conduction delay on the millisecond scale, so the analysis of temporal delay in the existing fNIRS-sEMG combination studies is relatively weak. Future trimodality studies combining high-frequency sEMG and EEG may better address this issue.

### 5.4 Limitations

Despite the above findings, several limitations of this study cannot be ignored.

There are some deficiencies in literature retrieval and screening in this review. The research process does not strictly follow the PRISMA-ScR systematic review retrieval and reporting standard, and the steps of literature screening, judgment criteria and exclusion basis are not systematic or transparent, which can easily lead to the omission of relevant research literature. Thus weakening the coverage breadth and rigor of the conclusions of this review.

At the conceptual level, cortex-muscle coherence is a classical analysis concept in the study of EEG-sEMG. With the gradual popularization of fNIRS-sEMG, the analysis of muscle-brain coupling using this combination is a derivative of the traditional concept of cortical-muscle coherence. Traditional corticomuscular coherence is mostly based on sEMG, EEG, and magnetoencephalography (MEG), and its signal acquisition and coupling analysis system has developed well. However, fNIRS-sEMG coupling relies on the blood-oxygen-level-dependent signal for evaluation, and there are essential differences in signal source, physiological connotation and analysis logic between the two paradigms. In this paper, there is a lack of distinction and comparison between the two types of coupling paradigms.

At the technical level, the short time resolution of fNIRS will directly restrict the validity of some coupling indicators. Coupling parameters with stringent requirements for signal sampling frequency and timing accuracy, such as phase synchronization and dynamic time-series correlation, are prone to calculation bias due to the limited update rate of the fNIRS signal, making it difficult to accurately capture the instantaneous dynamic coupling characteristics of neuromuscular activity. To solve this problem, the existing hardware upgrade scheme can effectively address it. On the one hand, a high-sampling-rate fNIRS device is used to improve the temporal resolution of blood-oxygen-signal acquisition. On the other hand, the hardware layout was optimized to improve temporal registration of fNIRS and sEMG signals by reducing the distance between the optical poles, thereby enhancing the accuracy of signal acquisition in local brain regions.

At the artifact-processing level, although fNIRS is not sensitive to motion artifacts, rapid head motion during vigorous exercise can still lead to signal aliasing and affect the accuracy of the hemodynamic model (11). Regarding the difficulty of feature decoupling, there is a complex, nonlinear relationship between cortical and muscle activation. Extracting pure coupling features from mixed signals remains a hot topic in current algorithm research.

At the level of clinical standardization, there is still a lack of a unified threshold for cortico-muscular coupling indicators that can be directly used for clinical diagnosis, and larger samples and multi-center verification are needed. Most of the studies included in this review were short-term observational studies, and some had sample sizes of fewer than 10 cases, so the demonstration of long-term effects and rigor remains insufficient. Future researchers can conduct long-term longitudinal follow-up studies, combined with a multi-center, large-sample experimental design, to dynamically monitor the remodeling of the central nervous circuit and the muscle regulatory network under different rehabilitation interventions and systematically interpret the characteristics of neural plasticity changes. High-quality clinical data are supplemented by long-term cohort studies to strengthen the theoretical framework and experimental basis in this field and to provide more robust, evidence-based support for the analysis of rehabilitation mechanisms, the optimization of intervention programs, and clinical applications.

### 5.5 Future perspectives

Multimodal physiological signal fusion is a key research hotspot in biomedicine and neuroscience. Based on the existing research paradigm of fNIRS-sEMG joint detection, multiple neuroimaging detection techniques, such as EEG and fMRI, can be further integrated (45) to overcome the limitations of a single detection method and construct a neuromuscular regulatory network model with more complete dimensions, more rigorous logic and wider coverage. To comprehensively analyze the synergistic mechanism of the central nervous system and the peripheral muscles.

At the level of clinical transformation and application of BCI, real-time decoding of muscle activation rules and motor regulation patterns by fNIRS-sEMG bimodal signals (46) can be used to find precise targets for physical therapy, such as transcranial magnetic therapy and achieve precise control of exoskeleton rehabilitation robots, intelligent prostheses and other auxiliary equipment. To promote the development of natural and intelligent human-computer interaction technology, and provide a new technical path for the rehabilitation intervention of people with motor dysfunction.

## 6 Conclusion

fNIRS-sEMG combined technology has become a powerful tool for studying the mechanism of muscle-brain coupling due to its advantages of anti-motion artifact, portability, non-invasiveness, and ease of use. It not only reveals the compensatory cortical activation and coupling dysfunction in patients with neurological diseases such as cerebral palsy and stroke, and people with chronic pain disorders, but also provides a new perspective on motor control strategies in healthy individuals. The author reviewed the above literature and found stable, repeatable functional coupling between specific cortical regions and specific muscle groups. Patients with neurological diseases show compensatory cortical activation with a dissociation pattern of “high activation, low coupling” and delayed activation time. With advances in data processing algorithms and the continued miniaturization of hardware, fNIRS-sEMG is expected to have greater clinical value for personalized rehabilitation evaluation, brain-computer interface control, and early disease diagnosis (47).

## Glossary

fNIRS: Functional Near-Infrared Spectroscopy
sEMG: Surface Electromyography
Hbo: Oxygenated hemoglobin
HbR: Deoxygenated hemoglobin
MDF: Median Frequency
MNF: Mean Frequency
DLPFC: Dorsolateral Prefrontal Cortex
M1: Primary Motor Cortex
PFC: Prefrontal Cortex
PMC: Premotor Cortex
S1: Primary Somatosensory Cortex
SMA: Supplementary Motor Area
OL: Occipital Lobe
SFC: Superior Frontal Cortex
MC: Motor Cortex
SAC: Somatosensory Association Cortex
CCI: Corticomuscular Coherence Index
Cv: Coefficient of Variation
ΔHbO: Change in Oxygenated Hemoglobin
fApEn: Fuzzy Approximate Entropy
LI: Laterality Index
MAV: Mean Absolute Value
RMS: Root Mean Square
SSI: Synergy Stability Index
TE: Transfer Entropy
r: Pearson Correlation Coefficient
CL-MC: Contralateral Motor Cortex
IMC: Ipsilateral Motor Cortex
CMC: Corticomuscular coherence
FC: Functional Connectivity
PSI: Phase Synchronization Index
CT: Contour Temporal Similarity
EC: Effective Connectivity
CC: Clustering Coefficient
NLE: Nodal Local Efficiency
MA: Mean Amplitude
AT: Activation Timing

## References

[1] Liu J Sheng Y Liu H. Corticomuscular coherence and its applications: a review. Front Hum Neurosci. (2019) 13:100. doi: 10.3389/fnhum.2019.00100, 30949041 PMC6435838

[2] Peng J Zikereya T Shao Z Shi K. The neuromechanical of beta-band corticomuscular coupling within the human motor system. Front Neurosci. (2024) 18:1441002. doi: 10.3389/fnins.2024.1441002, 39211436 PMC11358111

[3] Yang Y Dewald JPA van der Helm FCT Schouten AC. Unveiling neural coupling within the sensorimotor system: directionality and nonlinearity. Eur J Neurosci. (2018) 48:2407–15. doi: 10.1111/ejn.13692, 28887885 PMC6221113

[4] Bourguignon M Jousmäki V Dalal SS Jerbi K De Tiège X. Coupling between human brain activity and body movements: insights from non-invasive electromagnetic recordings. NeuroImage. (2019) 203:116177. doi: 10.1016/j.neuroimage.2019.116177, 31513941

[5] Witham CL Riddle CN Baker MR Baker SN. Contributions of descending and ascending pathways to corticomuscular coherence in humans. J Physiol. (2011) 589:3789–800. doi: 10.1113/jphysiol.2011.211045, 21624970 PMC3171886

[6] Almajidy RK Mankodiya K Abtahi M Hofmann UG. A newcomer's guide to functional near infrared spectroscopy experiments. IEEE Rev Biomed Eng. (2020) 13:292–308. doi: 10.1109/RBME.2019.2944351, 31634142

[7] Xie L Liu Y Gao Y Zhou J. Functional near-infrared spectroscopy in neurodegenerative disease: a review. Front Neurosci. (2024) 18:1469903. doi: 10.3389/fnins.2024.1469903, 39416953 PMC11479976

[8] Herold F Wiegel P Scholkmann F Thiers A Hamacher D Schega L. Functional near-infrared spectroscopy in movement science: a systematic review on cortical activity in postural and walking tasks. Neurophotonics. (2017) 4:041403. doi: 10.1117/1.NPh.4.4.041403, 28924563 PMC5538329

[9] Khan OA Rahman S Baduni K Modlesky CM. Assessment of cortical activity, functional connectivity, and neuroplasticity in cerebral palsy using functional near-infrared spectroscopy: a scoping review. Dev Med Child Neurol. (2025) 67:875–91. doi: 10.1111/dmcn.16238, 39963963 PMC12134447

[10] Merletti R Muceli S. Tutorial. Surface EMG detection in space and time: best practices. J Electromyogr Kinesiol. (2019) 49:102363. doi: 10.1016/j.jelekin.2019.102363, 31665683

[11] Wang X Li W Song R Ao D Hu H Li L. Corticomuscular coupling alterations during elbow isometric contraction correlated with clinical scores: an fNIRS-sEMG study in stroke survivors. IEEE Trans Neural Syst Rehabil Eng. (2025) 33:696–704. doi: 10.1109/TNSRE.2025.3535928, 40031336

[12] Seynaeve M Mantini D de Beukelaar TT. Electrophysiological approaches to understanding brain-muscle interactions during gait: a systematic review. Bioengineering (Basel). (2025) 12:471. doi: 10.3390/bioengineering12050471 40428090 PMC12108685

[13] Curzel F Tillmann B Ferreri L. Lights on music cognition: a systematic and critical review of fNIRS applications and future perspectives. Brain Cogn. (2024) 180:106200. doi: 10.1016/j.bandc.2024.106200, 38908228

[14] Alcan V Zinnuroğlu M. Current developments in surface electromyography. Turk J Med Sci. (2023) 53:1019–31. doi: 10.55730/1300-0144.5667, 38813041 PMC10763750

[15] Xu L Peri E Vullings R Rabotti C Van Dijk JP Mischi M. Comparative review of the algorithms for removal of electrocardiographic interference from trunk electromyography. Sensors (Basel). (2020) 20:4890. doi: 10.3390/s20174890, 32872470 PMC7506664

[16] Papagiannis GI Triantafyllou AI Roumpelakis IM Zampeli F Garyfallia Eleni P Koulouvaris P . Methodology of surface electromyography in gait analysis: review of the literature. J Med Eng Technol. (2019) 43:59–65. doi: 10.1080/03091902.2019.1609610, 31074312

[17] Savithri CN Priya E Rajasekar K. A machine learning approach to identify hand actions from single-channel sEMG signals. Biomed Tech (Berl). (2022) 67:89–103. doi: 10.1515/bmt-2021-0072, 35191277

[18] Chowdhury RH Reaz MB Ali MA Bakar AA Chellappan K Chang TG. Surface electromyography signal processing and classification techniques. Sensors. (2013) 13:12431–66. doi: 10.3390/s130912431, 24048337 PMC3821366

[19] Pinti P Scholkmann F Hamilton A Burgess P Tachtsidis I. Current status and issues regarding pre-processing of fNIRS neuroimaging data: an investigation of diverse signal filtering methods within a general linear model framework. Front Hum Neurosci. (2018) 12:505. doi: 10.3389/fnhum.2018.00505, 30687038 PMC6336925

[20] Guo Z McClelland VM Simeone O Mills KR Cvetkovic Z. Multiscale wavelet transfer entropy with application to corticomuscular coupling analysis. IEEE Trans Biomed Eng. (2022) 69:771–82. doi: 10.1109/TBME.2021.3104969 34398749

[21] Moll I Essers JMN Marcellis RGJ Senden RHJ Janssen-Potten YJM Vermeulen RJ . Lower limb muscle fatigue after uphill walking in children with unilateral spastic cerebral palsy. PLoS One. (2022) 17:e0278657. doi: 10.1371/journal.pone.0278657, 36473000 PMC9725134

[22] Sun J Jia T Lin PJ Li Z Ji L Li C. Multiscale canonical coherence for functional corticomuscular coupling analysis. IEEE J Biomed Health Inform. (2024) 28:812–22. doi: 10.1109/JBHI.2023.3332657 37963005

[23] Cheng C Liu T Zhang B Wu X Song Z Zhao Z . Effects of robot-assisted hand function therapy on brain functional mechanisms: a synchronized study using fNIRS and sEMG. Front Med (Lausanne). (2024) 11:1411616. doi: 10.3389/fmed.2024.1411616 39544380 PMC11560759

[24] Wei X Wei X Li Z Jamid S Wang H. Corticomuscular coupling alterations in subacute stroke patients: insights from fNIRS and sEMG. BMC Neurol. (2025) 26:45. doi: 10.1186/s12883-025-04589-4, 41436980 PMC12825247

[25] Yang Z Ye L Yang L Lu Q Yu A Bai D. Early screening of post-stroke fall risk: a simultaneous multimodal fNIRs-EMG study. CNS Neurosci Ther. (2024) 30:e70041. doi: 10.1111/cns.70041, 39315509 PMC11420627

[26] Ye S Tao L Gong S Ma Y Wu J Li W . Upper limb motor assessment for stroke with force, muscle activation and interhemispheric balance indices based on sEMG and fNIRS. Front Neurol. (2024) 15:1337230. doi: 10.3389/fneur.2024.1337230, 38694770 PMC11061400

[27] Hervey N Khan B Shagman L Tian F Delgado MR Tulchin-Francis K . Motion tracking and electromyography-assisted identification of mirror hand contributions to functional near-infrared spectroscopy images acquired during a finger-tapping task performed by children with cerebral palsy. Neurophotonics. (2014) 1:025009. doi: 10.1117/1.NPh.1.2.025009, 26157980 PMC4478941

[28] Zhou F Qi L Sun W Wang J. The lower limb coordination, brain activation during walking and their correlation in adolescents with spastic cerebral palsy: a pilot cross-sectional fNIRS study. Res Dev Disabil. (2026) 168:105197. doi: 10.1016/j.ridd.2025.105197, 41475314

[29] Jochumsen M Shafique M Hassan A Niazi IK. Movement intention detection in adolescents with cerebral palsy from single-trial EEG. J Neural Eng. (2018) 15:066030. doi: 10.1088/1741-2552/aae4b8, 30260322

[30] Fu R Ma J Zu Y Fang Y Li Y Wong AYL . Neuromuscular control mechanism during posture-cognition dual task in mild cognitive impairment: a concurrent fNIRS-sEMG-COP study. Geroscience. (2026). doi: 10.1007/s11357-026-02303-x, 42141268

[31] Wang X Luo Z Zhang M Zhao W Xie S Wong SF . The interaction between changes of muscle activation and cortical network dynamics during isometric elbow contraction: a sEMG and fNIRS study. Front Bioeng Biotechnol. (2023) 11:1176054. doi: 10.3389/fbioe.2023.1176054, 37180038 PMC10167054

[32] Li H Lin X Wu X. Dual-channel mechanism of groove music fused with tai chi to improve cognitive-emotional abilities in older adults based on a coupled fNIRS-EMG analysis: a randomized controlled study. Geroscience. (2025) 47:6583–97. doi: 10.1007/s11357-025-01811-6, 40721570 PMC12635002

[33] Jiajia Y Zengming H Nanhe L Xue C Yushu Z Yan L . The immediate effects of iTBS on the muscle activation pattern under challenging balance conditions in the patients with chronic low back pain: a preliminary study. Front Neurosci. (2023) 17:1135689. doi: 10.3389/fnins.2023.1135689, 36998734 PMC10045989

[34] Deng Q Jin G Lou X Ding Y Xu H Yang K . Differences in sensory-motor cortex activation patterns during level and stair walking in patients with knee osteoarthritis: protocol for a cross-sectional study. Front Aging Neurosci. (2025) 17:1589645. doi: 10.3389/fnagi.2025.1589645, 40458567 PMC12127309

[35] Trišins M Zdanovskis N Platkājis A Šneidere K Kostiks A Karelis G . Brodmann areas, V1 atlas and cognitive impairment: assessing cortical thickness for cognitive impairment diagnostics. Medicina (Kaunas). (2024) 60:587. doi: 10.3390/medicina60040587 38674233 PMC11052167

[36] Glasser MF Coalson TS Robinson EC Hacker CD Harwell J Yacoub E . A multi-modal parcellation of human cerebral cortex. Nature. (2016) 536:171–8. doi: 10.1038/nature18933, 27437579 PMC4990127

[37] Dai C Lin X Xue B Xi X Gao M Liu X . Correlation of bilateral M1 hand area excitability and overall functional recovery after spinal cord injury: protocol for a prospective cohort study. BMC Neurol. (2024) 24:213. doi: 10.1186/s12883-024-03705-0, 38909175 PMC11193300

[38] Han T Dai C Liang Y Lin X Gao M Liu X . PFC/M1 activation and excitability: a longitudinal cohort study on fatigue symptoms in healthcare workers post-COVID-19. J Transl Med. (2024) 22:720. doi: 10.1186/s12967-024-05319-z, 39103842 PMC11299412

[39] Liu L He J Nong F Huang Y Huang S Qin X . Changes in cortical activation during proprioceptive stimulation and galvanic vestibular stimulation in healthy individuals and individuals with post-stroke balance disorders: a functional near-infrared spectroscopy study. Neuroimage Clin. (2025) 47:103822. doi: 10.1016/j.nicl.2025.103822, 40499477 PMC12178929

[40] Wu X Xu M Sun W Lin X Xue B Ju F . The analgesic enhancing effects of coupling M1 and PMC rTMS on neuropathic pain after spinal cord injury: an fNIRS study. Pain Res Manag. (2026) 2026:4002703. doi: 10.1155/prm/4002703, 41623937 PMC12859385

[41] Yang J Zhang G Gao X Cheng X Hao Z Ma J . Role of dorsolateral prefrontal cortex during motor preparation on anticipatory postural adjustments. Brain Topogr. (2025) 38:44. doi: 10.1007/s10548-025-01120-3, 40413318

[42] Leuk JS Ng TH Goodwill AM Teo WP. Age-specific neural responses to SMA and M1 stimulation during implicit motor sequence learning: insights from a concurrent tDCS-fNIRS approach. Neuroscience. (2025) 577:240–51. doi: 10.1016/j.neuroscience.2025.05.002, 40345479

[43] Lin XM Xue YS Liu YH Hong R Xu WR Li Y . Multidimensional analysis of brain activation patterns in different motor therapies using functional near-infrared spectroscopy. Front Neurol. (2025) 16:1656369. doi: 10.3389/fneur.2025.1656369, 41383234 PMC12689357

[44] Roze E Dubacq C Welniarz Q. Corticospinal tract development, evolution, and skilled movements. Mov Disord. (2025) 40:1221–32. doi: 10.1002/mds.30199, 40277091 PMC12273621

[45] Khan H Naseer N Yazidi A Eide PK Hassan HW Mirtaheri P. Analysis of human gait using hybrid EEG-fNIRS-based BCI system: a review. Front Hum Neurosci. (2020) 14:613254. doi: 10.3389/fnhum.2020.613254, 33568979 PMC7868344

[46] Ahn S Jun SC. Multi-modal integration of EEG-fNIRS for brain-computer interfaces—current limitations and future directions. Front Hum Neurosci. (2017) 11:503. doi: 10.3389/fnhum.2017.00503, 29093673 PMC5651279

[47] Calabrò RS Calderone A Simoncini L Naro A Haughton LOS Quartarone A . The potential of robotics: a systematic review of neuroplastic changes following advanced lower limb rehabilitation in neurological disorders. Neurosci Biobehav Rev. (2026) 180:106459. doi: 10.1016/j.neubiorev.2025.106459, 41213447

